# Sex representation of editors, editorial boards, and authors of infectious diseases and healthcare epidemiology journals

**DOI:** 10.1017/ash.2023.346

**Published:** 2023-09-29

**Authors:** Aldo Barajas-Ochoa, Manuel Ramirez-Trejo, Aditee Dash, Jillian Raybould, Gonzalo Bearman

## Abstract

**Background:** Academic publishing is not exempt from potential structural disparities. We assessed the sex representation among the editors and on editorial boards by their level of influence in the decision of a manuscript of the leading journals focused on infectious diseases and healthcare epidemiology. We also explored whether the sex of the first or last author correlates with the sex of the editors in a convenience sample of these journals. **Methods:** In a cross-sectional study, the 40 top infectious disease journals (Scimago Journal and Country Rank) and 4 healthcare epidemiology journals were selected. The names and positions of the editorial members were extracted from the journal’s website, and a decision-making level was assigned (ie, editor-in-chief as level 1, board members as level 3). Next, the first and corresponding authors’ names of all 2019 research articles published in a convenience sample of 15 of these journals were retrieved for the second aim. A digital gallery was used to assign one of the binary denominations of woman or man based on the probability that a name was culturally given to a woman or man. Differences were determined by χ^2^ and linear regression. **Results:** Overall, 2,416 names were retrieved from the editorial boards of 44 journals; 799 (33%) were assigned as women and 1,617 (67%) as men. The decision-making level showed 70 (3%) at the editor-in-chief level, 756 (31%) at the associate editor level, and 1,600 (66%) as editorial board members. The frequency distribution of assigned gender by decision-making level showed 21 (30%) women and 49 (70%) men at the editor-in-chief level; 263 (35%) women and 493 (65%) men at the associate editor level; 515 (32%) women and 1,075 (68%) men at the editorial board level. Some journals showed an even sex distribution, such as *Clinical Infectious Disease* or *Microbiology Spectrum*. However, others were significantly unbalanced. We retrieved 2,725 articles from the convenience sample of infectious disease–focused journals. Women were the first authors in 1,373 (50%) and the last authors in 974 (35%). Editorial board sex composition and sex of authors showed no significant correlation. Trends between infectious disease–focused and healthcare epidemiology–focused journals were similar. **Conclusions:** Although the data showed uneven sex representation on the editorial boards of infectious disease–focused and healthcare epidemiology–focused journals, there is no apparent vertical segregation or influence on publishing by sex. A generational transition seems to be occurring in editorship and authorship in the field.

**Disclosures:** None

